# Sarcoidosis presenting as vocal cord palsy: an unusual presentation and literature review

**DOI:** 10.1002/rcr2.705

**Published:** 2020-12-23

**Authors:** Mohummad Hassan Raza Raja, Muhammad Abdullah Javed, Ali Bin Sarwar Zubairi

**Affiliations:** ^1^ Medical College Aga Khan University Karachi Pakistan; ^2^ Section of Pulmonary and Critical Care Medicine, Department of Medicine Aga Khan University Karachi Pakistan

**Keywords:** Cough, hoarseness of voice, respiratory structure and function, sarcoidosis, vocal cord paralysis

## Abstract

We report the case of a 60‐year‐old woman, presenting with left vocal cord paralysis (VCP). Diagnostic evaluation revealed sarcoidosis to be the underlying pathology. Computed tomography (CT) scans exhibited enlarged subcarinal, pretracheal, and prevascular lymph nodes as the possible mechanism of left VCP through compression of the left recurrent laryngeal nerve. Initial treatment with oral prednisolone and azathioprine led to remission of symptoms; however, tapering of dosage led to relapse of cough without any signs of VCP. The dosage of prednisolone was further boosted, leading to complete remission. A review of literature reveals 20 cases have been reported to date, with all but one involving the left vocal cord. This is the first reported case, with a relapse of the disease without a relapse of VCP, indicating the rarity of sarcoidosis‐associated VCP.

## Graphical abstract

We report the case of a 60‐year‐old woman, presenting with left vocal cord paralysis (VCP). She was diagnosed with sarcoidosis and treated with corticosteroids with significant improvement; however, tapering of steroid dosage led to relapse of symptoms, without a relapse of the vocal cord palsy. This is the first reported case, with a relapse of the disease without a relapse of VCP, indicating the rarity of sarcoidosis‐associated VCP.

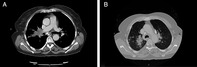

## Introduction

Sarcoidosis is a multisystemic disease of unknown aetiology which often involves multiple organs, most commonly the lungs and lymph nodes. We report an interesting case, which initially presented as hoarseness of voice accompanied with cough, weight loss, and enlarged mediastinal lymph nodes. Following the diagnosis on mediastinoscopic lymph node biopsy, the patient was started on corticosteroid therapy with significant improvement; however, tapering of steroid dosage led to relapse of symptoms, without a relapse of the vocal cord palsy.

## Case Report

A 60‐year‐old, non‐smoker, female, known case of hypertension, presented to the Ear, Nose & Throat (ENT) clinic with a history of hoarseness of voice, significant weight loss, and chronic cough for the past six weeks. The cough was dry initially, although at the time of presentation it turned productive of mucoid sputum. The coughing episodes were significantly more prevalent during night, and there was associated shortness of breath on exertion. She also reported a persistent globus sensation and difficulty in swallowing. There was no history of fever, night sweats, rheumatological symptoms, and chronic gastroesophageal reflux or post nasal drip symptoms. Furthermore, she reported positive exposure to biomass fuel during her childhood; however, exposure to pets was unremarkable. She was treated with montelukast and levofloxacin for suspected upper respiratory tract infection; however, this regimen did not improve her symptoms.

The patient's blood pressure measured 139/70 mmHg, pulse 76/min, temperature 37.1°C, respiratory rate of 17 breaths/min, and arterial oxygen saturation (SpO_2_) of 97%. Chest examination revealed bilateral vesicular breath sounds with no added sounds. The rest of the systemic and general examination was unremarkable.

A bronchoscopy performed outside our institution was unremarkable and a bronchoalveolar lavage (BAL) was negative for Acid‐Fast Bacilli (AFB) smear, Gene Xpert, and fungal cultures. The serum angiotensin‐converting enzyme (ACE) was 48 IU/L (reference range: 0–52/IU/L). Pulmonary functional test (PFT) demonstrated the presence of a significant restrictive pathology, with forced vital capacity (FVC) being 52% of the predicted value, forced expiratory volume in 1 sec (FEV_1_) 55% of predicted, and FEV_1_/FVC ratio being 77%.

**Figure 1:**
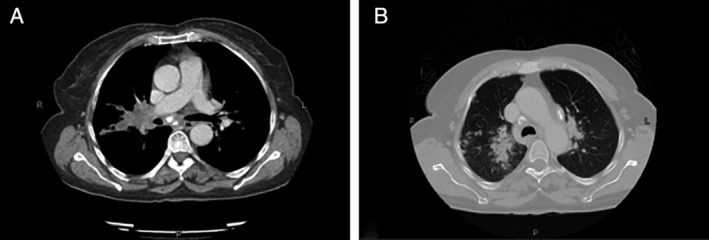
(A) Computed tomography (CT) scan of the chest mediastinal window displaying enlarged, calcified subcarinal lymph node. (B) Lung window showing right‐sided lung parenchymal infiltrates.

The patient underwent flexible fibre‐optic laryngoscopy which showed a fixed left vocal cord, with no lesion identified. A computed tomography (CT) scan with contrast of the thorax revealed bilateral perihilar ill‐defined opacification with multifocal scattered alveolar and nodular infiltrates. Enlarged mediastinal lymph nodes (prevascular, pretracheal, paracardiac, and subcarinal) with calcifications were also noted, with the largest lymph node in the subcarinal location, measuring approximately 12 mm in the short‐axis diameter (Fig. 1). There was no evidence of any extra‐thoracic lesions. A CT‐guided core lung biopsy revealed peri‐bronchial non‐necrotizing granulomatous lesions. A diagnosis of pulmonary sarcoidosis with left vocal cord paralysis (VCP) was made. Due to financial constraints, and reasonable clinical confidence in diagnosis, a fluorodeoxyglucose–positron emission tomography (FDG–PET) was not performed. The patient was started on oral prednisolone at 0.5 mg/kg/day.

She responded well to corticosteroid therapy with complete resolution of symptoms. A follow‐up PFT, two months post‐initiation of therapy, showed markedly improved pulmonary function with FVC being 71% of predicted, FEV_1_ 71% of predicted, and FEV_1_/FVC ratio being 81%. The corticosteroid therapy was tapered to a maintenance dose of 5 mg/day and the patient was started on azathioprine (100 mg/day).

The patient presented with relapse of symptoms of cough within few weeks of maintenance therapy. The patient denied any presence of dysphonia or hoarseness of voice. Repeat PFTs revealed marked deterioration, with FVC being 63% of predicted, FEV_1_ 57% of predicted, and FEV_1_/FVC ratio being 91% predicted. A high‐resolution CT (HRCT) chest revealed an ill‐defined bilateral, perihilar patchy opacification with multifocal scattered alveolar/nodular infiltrates demonstrating mild interval progression in the right perihilar region, along with enlarged calcified mediastinal lymph nodes (including the paracardiac lymph nodes). The patient was again started on 30 mg/day prednisolone and this resulted in significant improvement of symptoms. To prevent further relapse, another immunosuppressant (mycophenolate: 1000 mg/day) was added to the treatment regimen. Once the patient started showing clinical improvement, the corticosteroid therapy was gradually tapered to a maintenance dosage 2.5 mg/day.

## Discussion

Sarcoidosis is a complex, multisystem, granulomatous disease of unknown aetiology, ranging from asymptomatic to life‐threatening manifestations. It commonly involves the lung parenchyma, seen in up to 90% of patients [1]. Laryngeal manifestations of the disease remain rare, with reported incidence between 0.5% and 8.3% [2].

Among the potential laryngeal manifestations, supraglottic and subglottic involvement is usually implicated, with disease rarely affecting the true vocal cords [2]. Literature has shown three mechanisms involved with the development of VCP in sarcoidosis [3, 4, 5, 6, 7]. The first mechanism involves direct invasion of vocal cords by granulomatous tissue. This is considerably rare as the spread of sarcoidosis is mediated through the reticuloendothelial system and the true vocal cords are devoid of lymphatics. At present, only two cases of VCP due to direct invasion are reported in literature [2, 5]. In both cases, the direct invasion of granulomatous tissue affected both true vocal cords. Subglottic and supraglottic areas were also involved, suggesting contiguous spread to the true vocal cords from these areas. The second mechanism is a rare manifestation of sarcoidosis, known as neurosarcoidosis, whereby there is presence of polyneuritis of the cranial nerves. The third mechanism relates to the compression of the recurrent laryngeal nerves by enlarged mediastinal lymph nodes. This is the most common mechanism reported in 12 out of 19 cases of sarcoidosis with VCP [2, 4, 5]. The aetiology in our case also favours compressive lymphadenopathy due to enlarged paracardiac lymph nodes. Direct invasion of vocal cords was excluded in our case due to lack of any lesion or presence of granulation tissue visualized through flexible fibre‐optic laryngoscopy. Previous reports of neurosarcoidosis used magnetic resonance imaging (MRI) scans to diagnose neurosarcoidosis, which is the most sensitive modality [4]. In the current case, while neurosarcoidosis could not be excluded completely (due to lack of MRI stemming from financial concerns), this diagnosis remained unlikely due to the lack of any aberrant cranial neve or central nervous system (CNS) signs and symptoms or any CNS lesions visualized through CT imaging of the brain.

VCP may be unilateral or bilateral. Left VCP (LVCP) is most commonly reported, due to external compression of the recurrent laryngeal nerves. The left recurrent laryngeal nerve is longer than the right (12 vs. 6 cm) as it loops around the arch of the aorta and is more likely to be compressed by the mediastinal lymph nodes that are enlarged in sarcoidosis [6]. On the other hand, the right recurrent laryngeal nerve courses around the right subclavian artery and is less likely to be compressed. To date, only a single case has been reported with isolated right VCP (RVCP), with the suspected cause being compression of the right recurrent laryngeal nerve from the right para‐tracheal lymph nodes [8]. Among the three listed causes of VCP, only two, compressive lymphadenopathy and vagal neurosarcoidosis, have been implicated in unilateral VCP (Table 1).

**Table 1:** Reported cases of sarcoidosis‐associated unilateral VCP.

Author/year	Location	Age/gender	Vocal cord paralysed	Aetiology	Treatment	Response
Current case/2020	Karachi, Pakistan	60/Female	Left	Compressive lymphadenopathy	Prednisolone 0.5 mg/kg/day tapered over nine months to a maintenance dose of 2.5 mg/day	Persistence of lymphadenopathy Permanent resolution of LVCP after eight weeks
Wu et al./2019 [4]	New York, USA	51/Female	Left	Left vagal neurosarcoidosis	Steroids + left injection laryngoplasty with 2 mL of micronized dermis	Permanent resolution of LVCP
Mastan et al./2015 [6]	Wigan, UK	40/Female	Left	Left recurrent laryngeal nerve neurosarcoidosis	Prednisolone 40 mg/day tapered to 20 mg/day over six weeks	Persistence of LVCP due to stoppage of corticosteroid therapy after four weeks
Lop Gros et al./2014 [7]	Barcelona, Spain	35/Female	Left	Compressive lymphadenopathy	Prednisone 1 mg/kg/day for six months	Resolution of lymphadenopathy Persistence of LVCP after 24 weeks
Sekiguchi et al./2013 [9]	Minnesota, USA	60/Female	Left	Compressive lymphadenopathy	Prednisone 40 mg/day tapered over two years	Persistence of LVCP
Boyd et al. /2011 [8]	Virginia, USA	45/Male	Right	Compressive lymphadenopathy	Oral steroids for two months followed by paratracheal lymphadenectomy	Complete resolution of RVCP following paratracheal lymphadenectomy
Hughes and McGavin/1995 [10]	Plymouth, UK	74/Female	Left	Compressive lymphadenopathy	N/A	N/A
Jaffe et al./1994 [11]	Jerusalem, Israel	64/Male	Left	Compressive lymphadenopathy	Prednisone Initial dose of 60 mg/day tapered over eight months	Persistence of lymphadenopathy Persistence of LVCP after 32 weeks
Povedano Rodríguez et al./1992 [12]	N/A	53/Male	Left	Compressive lymphadenopathy	Prednisone	Complete resolution after six weeks
Tobias et al./1990 [13]	Los Angeles, USA	49/Male	Left	Compressive lymphadenopathy	Prednisone 40 mg/day	Resolution of lymphadenopathy Permanent Resolution of LVCP
el‐Kassimi et al. /1990 [14]	Riyadh, Saudi Arabia	45/Female	Left	Compressive lymphadenopathy	Prednisolone 40 mg/day	Resolution of lymphadenopathy Permanent resolution of LVCP after 1.5 weeks
Chijimatsu et al./1980 [15]	Tokyo, Japan	17/Male	Left	Compressive lymphadenopathy	Prednisolone 30 mg/day	Permanent resolution of LVCP after 2.5 weeks

LVCP, left VCP; RVCP, right VCP; VCP, vocal cord paralysis.

Bilateral VCP (BVCP) is less common than unilateral VCP, with eight reported cases in literature [4, 5]. BVCP is significantly more dangerous than unilateral VCP as it may lead to critical airway compromise, as reported by Hintze et al. [5]. Among the reported cases of BVCP, three were due to bilateral vagal neurosarcoidosis, three were due to compressive lymphadenopathy, and two were due to bilateral direct granulomatous invasion (Table 2).

**Table 2:** Reported cases of sarcoidosis‐associated BVCP.

Author/year	Location	Age/gender	Vocal cord paralysed	Aetiology	Treatment	Response
Wu et al./2019 [4]	New York, USA	48/Male	Both	Bilateral vagal neurosarcoidosis	Prednisone 50 mg/day followed by bilateral injection laryngoplasties with 2 mL of micronized dermis	Resolution of BVCP following laryngoplasty
Wu et al./2019 [4]	New York, USA	37/Male	Both	Bilateral vagal neurosarcoidosis	Injection laryngoplasty with 2 mL of micronized dermis, followed by left medialization laryngoplasty	Persistence of BVCP after 32 weeks
Hintze et al./2018 [5]	Arizona, USA	49/Female	Both	Bilateral direct granulomatous infiltration	Tracheostomy, Radiesse injection (Merz Aesthetics, Inc., Frankfurt, Germany), methylprednisolone 40 mg/day followed by prednisolone 40 mg/day tapered over six months	Persistence of BVCP after four years
Yamasue et al./2016 [3]	Oita, Japan	72/Female	Both	Bilateral vagal neurosarcoidosis	Prednisone (30 mg/day) tapered to 15 mg/day over four months	Complete resolution of BVCP after 16 weeks
Lop Gros et al./2014 [7]	Barcelona, Spain	61/Female	Both	Bilateral compressive lymphadenopathy	Prednisone 1 mg/kg/day for three months	Resolution of LVCP after 12 weeks, persistence of RVCP
Coffey et al./2009 [16]	Pennsylvania, USA	35/Male	Both	Bilateral compressive lymphadenopathy	Methylprednisolone 1 g/day followed by prednisone 40 mg/day	Complete resolution of BVCP after two weeks
Rupanagudi et al./2005 [2]	New York, USA	41/Female	Both	Bilateral direct granulomatous infiltration	Oral steroids for three months	Complete resolution of BVCP
Witt/2003 [17]	Delaware, USA	41/Female	Both	Cranial polyneuritis and compressive lymphadenopathy	Methylprednisolone 160 mg/day	Complete resolution of BVCP after one week

BVCP, bilateral VCP; LVCP, left VCP; RVCP, right VCP; VCP, vocal cord paralysis.

A review of literature reveals that the average age of presentation is 49, with 60% of patients being female (12 out of 20 cases). Left vocal cord involvement has been present in all but one case of sarcoidosis‐associated VCP (19 out of 20 cases).

For pulmonary sarcoidosis, systemic glucocorticoids remain the mainstay first‐line therapy [18]. As per consensus statement by the American Thoracic Society, European Respiratory Society, and World Association of Sarcoidosis and Other Granulomatous Disorders, the initial dose is 20–40 mg/day with eventual tapering, and discontinuation of therapy in 6–12 months [19]. In some cases, a maintenance dose is required for longer duration, as in our case to prevent relapse of symptoms. Even after corticosteroid therapy, vocal cord palsy may persist with resolution of symptoms with persistence of VCP being reported in seven out of 20 cases. In the seven cases of persistence, four were cases of LVCP, two were cases of BVCP, and one was a case of partially treated sarcoidosis associated BVCP where the RVCP persisted. In cases of recurrence or persistence of the vocal cord palsy, it is important for the physician to consider the aetiology of the vocal cord palsy. Surgical measures, such as lymphadenectomy, may be required to improve the quality of life. Boyd et al. reported a case of unilateral RVCP, which was cured following lymphadenectomy, as the compressive force on the right recurrent laryngeal nerve was relieved [8]. Less invasive measures such as laryngoplasty with injection of micronized dermis and speech and language therapy have also proven useful in improving the dysphonia [4, 6].

In our case, the patient initially responded well to the corticosteroid therapy, with complete resolution of symptoms. However, on maintenance dosage of prednisolone, there was relapse of disease. Interestingly, there was no relapse of the left vocal cord palsy. This is in spite of the repeat HRCT scan showing enlarged, calcified mediastinal lymph nodes (including paracardiac lymph nodes) that may have possibly compressed the left recurrent laryngeal nerve. This is particularly unusual as the presumptive cause of the initial LVCP had been compression of the left recurrent laryngeal nerve by enlarged mediastinal lymph nodes (primarily by the paracardiac lymph nodes), which remained persistent. In all previous cases of treated LVCP (due to compressive lymphadenopathy), resolution of lymphadenopathy leads to resolution of the LVCP.

In conclusion, we report a case of sarcoidosis associated with LVCP which resolved following oral corticosteroid therapy despite persistence of enlarged mediastinal lymph nodes and later relapse of the disease. The above literature demonstrates that sarcoidosis‐associated VCP remains an exceedingly rare presentation, it must be recognized and appropriately managed by clinicians.

### Disclosure Statement

Appropriate written informed consent was obtained for publication of this case report and accompanying images.

### Author Contribution Statement

MHRR: Data curation; writing—original draft; writing: review and editing. MAJ: Writing—original draft; writing—review and editing. ABSZ: Conceptualization; supervision; writing—review and editing.
